# Computer-aided three-dimensional assessment of periodontal healing distal to the mandibular second molar after coronectomy of the mandibular third molar: a prospective study

**DOI:** 10.1186/s12903-020-01250-z

**Published:** 2020-09-24

**Authors:** Z. Y. Yan, Y. Tan, X. Y. Xie, W. He, C. B. Guo, N. H. Cui

**Affiliations:** 1 Department of Oral and Maxillofacial Surgery, Peking University School and Hospital of Stomatology, 22 South Street Zhong Guan Cun, Haidian District, Beijing, 100081 China; 2 Department of Medical Statistics, Peking University Clinical Research Institute, Beijing, China; 3 Department of Oral and Maxillofacial Radiology, Peking University School and Hospital of Stomatology, Beijing, China

**Keywords:** Coronectomy, Computer-aided 3D assessment, Distal intra-bony defect, Periodontal healing, Re-contact, Volume of bone regenerated

## Abstract

**Background:**

The periodontal healing distal to the mandibular second molar (M2M) after coronectomy of the M3M has shown controversial results. We aimed to combine a digital method with cone-beam computed tomography (CBCT) and estimate periodontal healing of M2M after M3M coronectomy. An accurate and stable indicator in three dimensions was also explored tentatively.

**Methods:**

Patients with a M3M in contact with the inferior alveolar canal were included. CBCT was applied immediately after coronectomy (baseline) and 6-months later. Data were investigated with digital software for registration. Previously reported and coronectomy-related factors were included for univariate and multivariate analyses.

**Results:**

A total of 181 patients (213 M3Ms) completed 6-month follow-up. Significant reduction in the distal intra-bony defect (DBD) depth of the M2M was shown (1.28 ± 1.24 mm, *P* < 0.001). DBD depth of the M2M at baseline was the most influential factor (*r* = 0.59), followed by preoperative M3M condition, age, rotation and migration of the root complex. Remaining enamel (OR = 6.93) and small retromolar space (0.67) contributed to re-contact of the root complex and M2M. Bone volume regenerated in the distal 2 mm was associated significantly with DBD-depth reduction (*r* = 0.74, *P* < 0.001).

**Conclusions:**

Bone volume regenerated in the distal 2 mm of the M2M denoted stability of distal periodontal healing of the M2M. DBD depth at baseline was the most influential factor for healing of a DBD of the M2M after M3M coronectomy. The remaining enamel and a small retromolar space could contribute to re-contact of the root complex and the M2M.

**Trial registration:**

China Clinical Trial Center, ChiCTR1800014862. Registered 10 February 2018,

## Background

“Coronectomy” is an alternative to total extraction of the mandibular third molar (M3M) to protect the inferior alveolar nerve (IAN) [1, 2]. Coronectomy is applied mainly to a deeply impacted M3M vulnerable to a distal intra-bony defect (DBD) of the mandibular second molar (M2M) after M3M surgery [3]. Just as in total extraction, the prognosis of a DBD continues to challenge clinicians [4]. Most studies have claimed that a DBD of the M2M remains unchanged or improves [5–7] but several other studies have suggested that this condition worsens after M3M surgery [8]. The reason for this difference could be because of an unsatisfactory design of the clinical study [9, 10] and/or limitations in periodontal-probing factors (pocket depth and attachment loss).

Most studies on M2M conditions after M3M surgery have focused only on the indicators of periodontal probing [11, 12]. In fact, the accuracy of subjective recording would be affected by unsatisfactory vision in the posterior oral cavity and variable location of the gingival margin [13–15]. Conversely, indicators of periodontal probing cannot eliminate interference due to repair of the long-junctional epithelium, which is characterized as repair or very limited regeneration [16].

Compared with indicators of periodontal probing, bone levels on radiography seem more stable. Faria used the cementoenamel junction (CEJ) as a reference to assess the DBD of the M2M on digital periapical radiographs, and obtained a relatively stable result [10]. However, due to the overlap of two-dimensional (2D) imaging, changes in bone height on the buccal and lingual sides could not be measured separately [17]. In addition, changes in bone height could barely reflect the possibility of further bone resorption. An accurate and predictable indicator in three dimensions is needed.

Recently, a combination of 3D cone-beam computed tomography (CBCT) data and digital software has enabled the reconstruction and registration of digital models of tooth or bone. This approach has provided excellent reproducibility and accuracy for comparative analyses [18, 19]. Guidance of implant surgery [20] and navigation of oral and maxillofacial surgery [21] can employ this new method.

We used a digital method to evaluate the DBD prognosis of the M2M after M3M coronectomy. Previously reported factors and coronectomy-related factors were included. In addition, an accurate and stable indicator in three dimensions was explored tentatively.

## Methods

This was a single-arm and single-blinded study. It is also part of an ongoing study on the long-term changes in the M3M after coronectomy [2]. Ethical approval of the study protocol was granted by the Biomedical Ethics Committee of Peking University Hospital of Stomatology (PKUSSIRB-201736080) in Beijing, China. This study was conducted in accordance with the Helsinki Declaration of 1975, as revised in 2013 and registered in the China Clinical Trial Center (ChiCTR1800014862). All patients provided written informed consent to participate in this study.

### Eligibility

From 2018 to 2019, patients who accepted coronectomy of an impacted M3M were recruited from the Department of Oral & Maxillofacial Surgery of Peking University School and Hospital of Stomatology.

Inclusion criteria were: (i) healthy males and females aged 18–40 years; (ii) a M3M impacting the inferior alveolar canal, as shown by preoperative CBCT; (iii) the patient could tolerate surgery.

Exclusion criteria were: (i) local susceptible factors such as caries, cystic/neoplastic conditions around the M3M; (ii) periodontal condition of the M2M: mesial alveolar bone resorption > 1/3 of root length, loose tooth, or smoking > 10 cigarettes per day [22]; (iii) general systemic disease contributing to infection (e.g., diabetes mellitus, immunodeficiency); (iv) history of radiotherapy or chemotherapy; (v) pregnancy.

The sample size for this study was calculated using SAS v8.3 (SAS Institute, Cary, NC, USA). The DBD depth of M2M before M3M coronectomy has been reported as 6.1 ± 2.8 mm [14]. To observe a difference of 0.5 mm in postoperative radiographs with a power of 90% at the 5% significance level, the sample size should be ≥137 subjects. Considering an anticipated dropout rate of 20%, a sample size of 172 subjects was necessary.

### Surgical procedure and imaging

During coronectomy, we applied a crown section 1–2 mm below the CEJ. The root surface was trimmed to ≥3 mm below the alveolar crest [1, 23]. After routine root planing of the M2M, debridement and irrigation, the wound was sutured. All surgical procedures were undertaken by the same senior surgeon. Neither antibiotics nor analgesic were administered.

CBCT was done twice: the day after surgery (baseline) and 6 months postoperatively (Po.6 m). A NewTom™ CBCT system (Quantitative Radiology, Verona, Italy) was applied to scan the region of interest. The imaging parameters were 110 kVp with a voxel size of 0.2 mm and field of view of 8 cm × 12 cm. The mA value was regulated automatically according to head size of each patient. The exposure time was 18-s each.

CBCT examination was carried out mainly to follow-up the condition of the M3M root complex after coronectomy [15, 23, 24]. Those data and analyses will be summarized in another report.

### Construction and measurement using the digital model

#### Registration and deviation

**Fig. 1:**
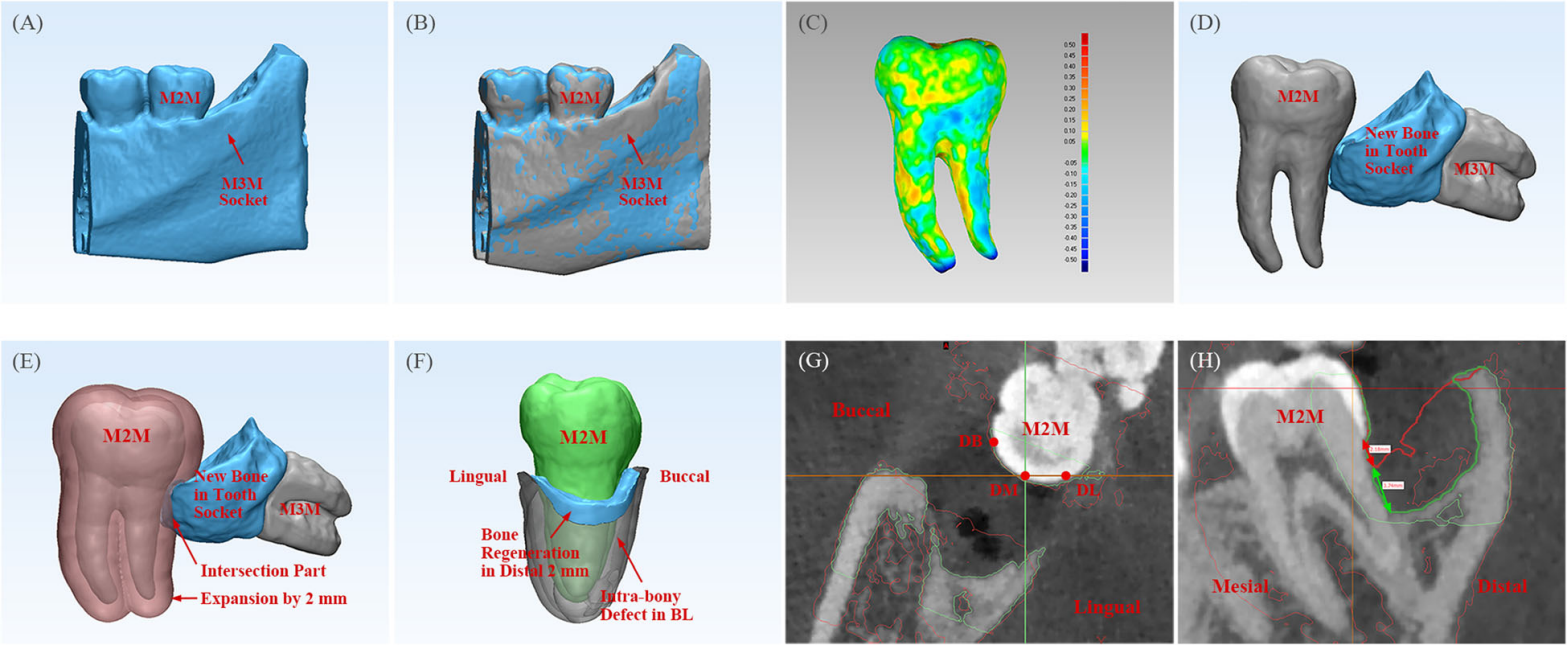
Construction and measurement using the digital model. **a** Construction of a digital model of an extraction socket. **b** Registration of a model of an extraction socket at different times (BL model in gray, Po.6 m model in blue). **c** 3D color map of the geometric deviation of the M2M at different times: the deviation range was set between −0.5 mm and 0.5 mm. The map started with blue (negative-error area), then passed through green (near no-error area), and ended with red (positive-error area). **d** Volume of total bone regenerated in a tooth socket (blue model). **e** Border of the M2M was expanded by 2 mm (transparent pink). Bone regeneration in the distal 2 mm of the M2M was determined as the interaction part of bone regenerated in the tooth socket (blue model) and expanded 2 mm of the M2M (transparent pink). **f** The DBD of the M2M at BL (transparent gray) and bone regeneration in the distal 2 mm of the M2M (blue model) could be observed visually. **g** Selection of DB, DM and DL sites of the M2M. **h** Measurement of the DBD depth of the M2M. Extraction-socket model at BL (bone margin is described as a green line) and Po.6 m (bone margin is described as a red line) was registered. The DBD depth at Po.6 m (red arrow) and reduction in the DBD depth (green arrow) was assessed, respectively. DBD = distal intra-bony defect

CBCT data in Digital Imaging and Communications in Medicine format at baseline and Po.6 m were imported into Mimics™ 19.0 (Materialise Dental, Leuven, Belgium). Digital models of mandibular bone, extraction socket (a digital model of hard tissue containing the entire extraction socket), the M2M, and root complex of the M3M after coronectomy were segmented and saved in stereolithography file format for further analyses in GeoMagic™ Studio 12 (3D Systems, Rock Hill, SC, USA) [19]. Models of mandible bone with clear anatomic markers were applied for registration according to the “Best-Fit Alignment” algorithm [25, 26]. Hence, the relative migration and variation at two times (baseline and Po.6 m) could be visualized directly. The geometric deviation of the M2M at different times was calculated according to the “Deviation” algorithm. Mean ± SD values were recorded and the distribution of distance shown on a graphical color map [19] (Fig. 1a–c).

#### Bone regeneration

The volume of bone regeneration and variation in the DBD depth (distance from the CEJ to the bottom of the osseous defect) were measured respectively.

##### Total volume of bone regenerated in the tooth socket

A new digital model was calculated using the “Boolean” algorithm to subtract the extraction-socket model at different times. It was assumed that there was no change in hard tissue surrounding the tooth socket in the 6 months after surgery, except for bone regeneration in the tooth socket. Hence, the difference between two models for the extraction socket could be regarded as the total volume of bone regenerated in the tooth socket (Fig. 1d).

##### Volume of bone regenerated in the distal 2 mm of the M2M

The digital model of the M2M was imported into 3-matic Medical 11.0 (Materialise Dental) and its border was expanded by 2 mm in three dimensions according to the “Wrap” algorithm. The interaction part of this new model and the model calculated above (total volume of bone regenerated in the tooth socket) was regarded as new bone regenerated in the distal 2 mm of the M2M (Fig. 1e, f). The reason why “2 mm” was chosen is explained in the Discussion section.

##### Variation in DBD depth of the M2M

Digital models of the extraction socket at different times, which had already been registered in GeoMagic, were imported into ProPlan™ CMF (Materialize). The change in DBD depth was measured visually at the site of the disto-buccal (DB) axial angle and disto-lingual (DL) axial angle of the M2M. The disto-middle (DM) depth was measured pass the midpoint of the distal marginal ridge (DMR) of the M2M (Fig. 1g, h).

### Preoperative and postoperative variables

All involved variables were measured and recorded by the same resident surgeon. To improve the accuracy of statistical analyses, the data were then transferred to other co-authors blinded to the study protocol for further analyses independently.

#### Demographic characteristics

The demographic characteristics were age and sex.

#### M3M-related factors

The M3M-related factors were: (i) Pell & Gregory’s depth of impaction; (ii) angulation of the M3M (the intersection angle of the M2M and M3M in the longitudinal axis was measured); (iii) retromolar space (RMS; calculated from the most distal point of the M2M to the anterior border of the ramus) [27].

#### M2M-related factors

There were four M2M-related factors. The first was DBD depth (baseline and Po.6 m). The second was the number of sites with DBD depth ≥ 4 mm. The third factor was the probing depth (PD). This was measured with a periodontal probe (Hu-Friedy, Chicago, IL, USA) at DL, DM and DB sites. The DMR of the M2M was used as a stable reference instead of variable free gingival margins. Postoperatively, the PD was measured before wound suture and recorded as distance from the DMR to the bottom of the DBD. At Po.6 m, the PD was measured at a manual pressure of approximately 20–30 g and recorded as the distance from the DMR to the bottom of the gingival sulcus or pocket. The fourth factor was external root resorption.

#### Coronectomy-related factors

The coronectomy-related factors were: (i) distance of root migration; (ii) angle of root rotation; (iii) remaining enamel of the root complex; (iv) thickness of the calcification bridge (radioresistant bone-like structure above the root section): it was measured at the center of the root fragment; (v) eruption status of the root complex: soft-tissue coverage (root section was completely covered with soft tissue or partially by bone) and bony impaction (root section was completely covered with bone). (vi) total volume of bone regeneration in the tooth socket.

#### Outcome parameters

The outcome parameters were: (i) changes in the DBD depth; (ii) changes in the PD; (iii) volume of bone regenerated in the distal 2 mm of the M2M; (iv) whether the root complex re-contacted the distal surface of the M2M.

### Statistical analyses

Statistical analyses were carried out using SPSS v21.0 (IBM, Armonk, NY, USA). Changes in the DBD depth and PD were analyzed using the paired *t*-test. Changes at sites with DBD depth ≥ 4 mm were analyzed using the chi-square test.

Outcome parameters were classified as continuous variables (changes in the DBD depth and PD, volume of bone regenerated in the distal 2 mm of the M2M) and binary variables (root complex re-contacted at the distal surface of the M2M). For continuous variables, Kendall’s tau-b correlation coefficient was applied as univariate analysis, followed by multiple linear regression as multivariate analysis. Chi-square tests, Mann–Whitney *U*-tests and binary logistic regression analysis were applied to binary variables. *P*<0.05 was considered significant.

## Results

A total of 220 patients (coronectomies of 259 impacted M3Ms) were evaluated. However, 213 M3Ms of 181 patients (72 males and 109 females) completed follow-up at Po.6 m. The age (mean ± SD) of the study cohort was 27.03 ± 4.84 years.

The mean change in the DBD depth of the M2M (mean of DB, DM, DL sites) and volume of bone regenerated in the distal 2 mm of the M2M was 1.28 ± 1.24 mm and 39.75 ± 26.03 mm^3^, respectively. The deviation of the digital registration was 0.01 ± 0.25 mm. Besides postoperative pain for a few days, all participants were asymptomatic at Po.6 m.

**Fig. 2:**
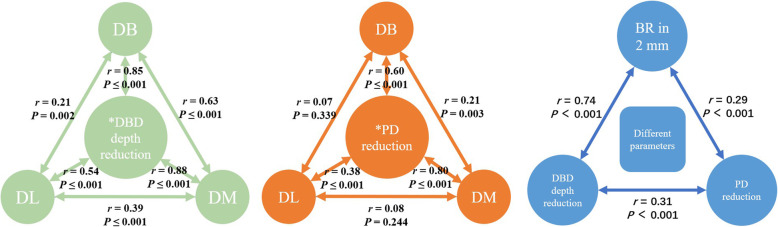
Correlation between different sites and different parameters. DB = disto-buccal; DM = disto-middle; DL = disto-lingual; DBD = distal intra-bony defect; BR in 2 mm = volume of bone regenerated in the distal 2 mm of M2M

Although, no significant difference was found at baseline (4.08 ± 1.77 vs. 4.45 ± 1.56 mm) (*P* = 0.105), the reduction in the DBD depth in patients younger than 25 years was significantly larger than that in their older counterparts at Po.6 m (1.55 ± 1.35 vs. 1.07 ± 1.10 mm) (*P* = 0.004). A significant decrease in the PD, DBD depth, and number of sites with DBD depth ≥ 4 mm was found at the distal sites (DB, DM, DL) of the M2M (*P* ≤ 0.001) (Table 1). The correlation among periodontal variations at DB, DM and DL sites is shown in Fig. 2. The mean ± SD of reduction in the PD and DBD depth, which was related significantly to variations at all distal sites (DB, DM, DL) of the M2M (*P* ≤ 0.001), was applied in further statistical analyses.

**Table 1:** Variation in the PD and DBD depth between BL and Po.6 m and difference between them

	DBD depth ≥ 4 mm			DBD depth (mm)			PD (mm)			PD reduction vs DBD depth reduction (mm)			
	BL	Po.6 m	***P***-value	BL	Po.6 m	***P***-value	BL	Po.6 m	***P***-value	PD reduction	DBD depth reduction	Difference	***P***-value
	%	%		Mean ± SD	Mean ± SD		Mean ± SD	Mean ± SD		Mean ± SD	Mean ± SD	Mean ± SD	
**DL**	18.31	6.10	**0.000**	2.70 ± 1.86	1.91 ± 1.19	**0.000**	6.33 ± 2.07	5.67 ± 1.18	**0.000**	0.61 ± 2.01	0.79 ± 1.38	− 0.21 ± 2.36	**NS**
**DM**	63.38	27.70	**0.000**	4.72 ± 2.17	3.18 ± 1.57	**0.000**	11.25 ± 3.70	4.57 ± 1.34	**0.000**	6.62 ± 3.88	1.55 ± 1.66	5.03 ± 4.00	**0.000**
**DB**	72.77	45.07	**0.000**	5.43 ± 2.29	3.95 ± 1.84	**0.000**	13.92 ± 2.74	6.94 ± 2.13	**0.000**	6.94 ± 3.04	1.50 ± 1.59	5.38 ± 2.84	**0.000**
**Mean value**	51.49	26.29	**0.000**	4.28 ± 1.66	3.01 ± 1.28	**0.000**	10.50 ± 1.72	5.73 ± 1.27	**0.000**	4.72 ± 1.86	1.28 ± 1.24	3.38 ± 1.84	**0.000**

*DBD* Distal intra-bony defect. PD at BL was the distance from the distal marginal ridge of the M2M to the bottom of the DBD. PD at Po.6 m was the distance from the distal marginal ridge of the M2M to the junctional epithelium. “NS” denotes “no significant difference”

Kendall’s tau-b correlation coefficient was applied to evaluate influential factors (Table 2). The DBD depth of the M2M at baseline was the factor (correlation coefficient (*r*) = 0.59) that was most influential to a reduction in the DBD depth at Po.6 m, followed by the preoperative M3M condition (≥0.20), age (*−* 0.23) as well as the rotation status (0.19) and eruption status (0.27) of the root complex (*P* ≤ 0.003). Besides, the DBD depth of the M2M at baseline was associated significantly with the preoperative depth of M3M impaction (*r* = 0.15), angulation of the M3M (0.40) and DBD depth of the M2M at Po.6 m (0.67) (*P* ≤ 0.031). The RMS was related negatively with the preoperative depth of M3M impaction (*r* = − 0.28) (*P* ≤ 0.001). Similar influences were found for PD reduction and the volume of bone regenerated in the distal 2 mm of the M2M. Also, a level of correlation was shown between reduction in the DBD depth and the volume of bone regenerated in the distal 2 mm of the M2M (*r* = 0.74, *P* ≤ 0.001) (Fig. 2).

**Table 2:** Correlation analyses of previously reported and coronectomy-related factors

			M3M condition			M2M condition		Coronectomy-related factors				
		Age	P&G‘s depth of impaction	Angulation of M3M	RMS	DBD depth at BL	ERR	Enamel remains	Root migration	Root rotation	Total volume of bone regenerated in tooth socket	Thickness of calcification bridge above root section
**DBD depth reduction**	*r*	−0.23	0.20	0.37	−0.36	0.59	−0.08	− 0.03	0.01	0.19	−0.01	0.27
	**P-value**	**0.001**	**0.003**	**0.000**	**0.000**	**0.000**	**NS**	**NS**	**NS**	**0.005**	**NS**	**0.000**
**PD reduction**	*r*	−0.09	0.07	0.28	−0.26	0.17	−0.01	− 0.06	0.02	0.06	−0.03	0.30
	**P-value**	**0.187**	**0.309**	**0.000**	**0.000**	**0.014**	**NS**	**NS**	**NS**	**NS**	**NS**	**0.000**
**Volume of bone regenerated in distal 2 mm**	*r*	−0.21	0.28	0.43	−0.44	0.42	−0.04	− 0.02	0.09	0.24	0.20	0.25
	**P-value**	**0.002**	**0.000**	**0.000**	**0.000**	**0.000**	**NS**	**NS**	**NS**	**0.000**	**0.004**	**0.000**

*DBD* Distal intra-bony defect, *RMS* Retromolar space, *ERR* External root resorption, *r* correlation coefficient; “NS” denotes “no significant difference”

Multiple linear regression models were established for factors influencing reduction in the DBD depth (adjusted R^2^ = 0.592) and volume of bone regenerated in the distal 2 mm of the M2M (adjusted R^2^ = 0.417) (Table 3). Both factors were associated significantly with age, the RMS, the DBD depth at baseline, as well as the rotation and eruption status of the root complex at Po.6 m (*P* ≤ 0.020). The total volume of bone regenerated in the tooth socket also influenced the volume of bone regenerated in the distal 2 mm of the M2M (coefficient: 0.14, *P* = 0.014). Furthermore, the RMS (odds ratio (OR) = 0.67) and remaining enamel of the M3M (OR = 6.93) were significant factors influencing re-contact of the root complex with the M2M (19 in 213 M3Ms) (*P* ≤ 0.037).

**Table 3:** Factors influencing bone variation and re-contact of the root complex and M2M

Multiple linear regression	Variables	B	Coef	P
**DBD depth redution**			**Adjusted R**^**2**^ **= 0.592**	
	Age	−0.07	−0.28	**0.000**
	RMS	−0.07	−0.13	**0.010**
	DBD depth at BL	0.49	0.66	**0.000**
	Rotation of root complex	0.02	0.12	**0.020**
	Eruption status of root complex	0.50	0.19	**0.000**
**Volume of bone regenerated in distal 2 mm**			**Adjusted R**^**2**^ **= 0.417**	
	Age	−1.21	−0.23	**0.000**
	RMS	−1.76	−0.17	**0.009**
	DBD depth at BL	5.69	0.36	**0.000**
	Rotation of root complex	0.52	0.14	**0.009**
	Total volume of bone regenerated in tooth socket	0.02	0.14	**0.014**
	Eruption status of root complex	9.64	0.18	**0.001**
**Binary logistic regression**	**Variables**	**OR**	**95%CL**	**P**
**Recontact of root complex and distal root surface of M2M**				
	RMS	0.67	0.46–0.97	**0.035**
	Enamel remains	6.93	1.13–42.58	**0.037**

*DBD* Distal intra-bony defect, *RMS* Retromolar space. “NS” denotes “no significant difference”

PD measurement was not the distance from free gingival reduction to the bottom of the gingival sulcus or pocket. The difference between PD reduction and reduction in the DBD depth could represent the thickness of soft tissue above the bone defect. The regression model could better interpret PD reduction when soft-tissue variation was included (adjusted R^2^ = 0.798) than when hard-tissue variation was included (0.618) (Table 4).

**Table 4:** Different multiple linear regression models for PD reduction

Independent factors included	Variables	B	Coef	P
**Baseline factors**	**Adjusted R**^**2**^ **= 0.576**			
	Age	−0.05	−0.13	**0.006**
	DBD depth at BL	−0.07	−0.07	**NS**
	PD at BL	0.85	0.78	**0.000**
**Baseline factors + hard tissue variation**	**Adjusted R**^**2**^ **= 0.618**			
	Age	−0.01	−0.03	**NS**
	DBD depth at BL	−0.33	−0.30	**0.000**
	PD at BL	0.86	0.79	**0.000**
	^***a***^***DBD depth reduction***	0.49	0.33	**0.000**
**Baseline factors + soft tissue variation**	**Adjusted R**^**2**^ **= 0.798**			
	Age	−0.07	−0.19	**0.000**
	DBD depth at BL	0.37	0.34	**0.000**
	PD at BL	0.20	0.19	**0.000**
	***# Difference between PD reduction and DBD depth reduction***	0.73	0.73	**0.000**

*DBD* Distal intra-bony defect, *RMS* Retromolar space. “NS” denotes “no significant difference”

Note: ^a^The DBD depth was regarded to be a factor related hard-tissue variation. #The difference between PD reduction and reduction in DBD depth was regarded to be a factor related to soft-tissue variation

## Discussion

The prognosis of a DBD of the M2M after M3M coronectomy has not been studied systematically and accurately previously. Only the effect of root migration and short-term complications of DBD variations have been analyzed with overlapped 2D radiography [14]. The influences of rotation, eruption status, and re-contact with adjacent teeth of the M3M root complex have been ignored, which are important and common coronectomy-related cofactors [28]. In addition, Vignudelli observed 4 ± 4 mm of bone regeneration 9 months after coronectomy, and claimed it was comparable with guided tissue regeneration after M3M extraction. They used a value of 3.59 ± 1.14 mm reported by Hassan [13] as a comparison. A new study with three-dimensional (3D) measurements should be applied to validate these results.

We combined digital technology and CBCT (which overcame the limitation of periodontal probing and 2D radiography) to measure bone-level changes in three dimensions accurately. Our main finding was that periodontal healing of the M2M after M3M coronectomy would not be disturbed by the remaining root complex. The volume of bone regenerated in the distal 2 mm of the M2M was an effective indicator in three dimensions.

After registration of CBCT at baseline and Po.6 m, digital technology showed a significantly lower deviation (0.01 ± 0.25 mm) than that using 2D radiography (±0.5 mm) [10]. Eliminating overlap in 2D radiography, all distal sites (DL, DM, DB) of the M2M were assessed and a significant difference in the PD and DBD depth was found (*P* ≤ 0.001) (Table 1). Po.6 m was chosen because the major part of periodontal healing occurs within 3 months after surgery [9]. The mean reduction in the DBD depth was 1.28 ± 1.24 mm, which is slightly higher than that documented in other studies (0.62–1.25 mm) [4, 6, 13]. Hence, the root complex did not disturb periodontal healing of the M2M. The change in the DBD depth (*r* ≥ 0.54) and PD (*r* ≥ 0.38) in all sites was corelated significantly with the mean value (Fig. 2). It was acceptable to use the mean value in subsequent statistical analyses.

The factors influencing periodontal healing of the M2M reported previously were analyzed in the present study. No significant difference was found between the DBD depth in patients younger than 25 years (4.08 ± 1.77 mm) and those older than 25 years (4.45 ± 1.56 mm) (*P* = 0.105). However, 6 months later, patients younger than 25 years (1.55 ± 1.35 mm) obtained a greater reduction in the DBD than their older counterparts (1.07 ± 1.10 mm) (*P* = 0.045). Correlation and regression analyses also showed a negative relationship between age and DBD reduction, and these observations were consistent with data from various studies [7, 29]. The original DBD depth has been reported to be a risk factor for periodontal healing of the M2M [7]. In the present study, a close correlation was shown between baseline and Po.6 m (*r* = 0.67, *P* ≤ 0.001) for DBD depth. Besides, the higher DBD in our study (5.43 ± 2.29 mm at baseline, 72.8 to 45.1%) explains why the decrease in the number of DB sites of DBD depth ≥ 4 mm was less than that reported in the study by Faria (4.54 ± 1.87 mm at baseline, 76.9 to 15.3%) [10]. However, the DBD depth at baseline showed a positive correlation with DBD reduction, with the highest correlation coefficients being reported among all the factors evaluated in our study (*r* = 0.59, *P* ≤ 0.001). These data suggested that compensatory bone formation would occur in patients with a large DBD depth at baseline, but their prognosis would not be better than that of patients with small DBD depth at baseline. These cases of coronectomy are important because the deeper the impaction of M3Ms preoperatively, the larger was the DBD depth at baseline. Similarly, deep impaction and large angulation of the M3M could contribute to a large DBD depth at baseline, as shown previously [3, 10, 30], and are related positively with DBD reduction (*r* ≥ 0.20, *P* ≤ 0.003).

Some factors related to coronectomy and periodontal healing have not been studied before. The RMS reflects whether the width for M3M eruption is sufficient [27]. The RMS was corelated negatively with the depth of M3M impaction (*r* = − 0.28, *P* ≤ 0.001). Significant correlation was observed between the RMS and reduction in DBD depth (*r* = − 0.36, *P* ≤ 0.001). Thus, it is reasonable to assume that, after coronectomy of the M3M, patients with a small RMS could obtain a narrow and deep pocket distal to the M2M [16]. Sculean reported that that a narrow and deep pocket would gain more bone than a wide and shallow defect. Hence, a patient with a small RMS could gain more bone than a patient with a large RMS. Rotation of the root complex can be observed in 65% of patients after coronectomy [28]. The positive relationship between root rotation and reduction in DBD depth has been considered to be orthodontic traction [31]. A bone-embedded root complex regenerated more bone distal to the M2M than that observed for soft tissue-covered roots (*r* = 0.27, *P* ≤ 0.001), data that are in accordance with results from a study by Yeung [23]. A correlation was not found between reduction in DBD depth and root migration, as reported previously [14, 31]. Analyses of the coronectomy-related factors mentioned above suggested that the remaining root complex did not disturb periodontal healing of the M2M. Re-contact of the root complex and distal surface of the M2M was a potential risk factor for the periodontal condition of the M2M; it caused two in five patients to undergo secondary surgery after coronectomy in a study by Monaco [32]. Meanwhile, a M3M with a complete root apex can erupt upon application of mechanical force to a M2M [33] and increase the risk of external root resorption [34]. Surgeons should note that the remaining enamel increased the risk of re-contact by 5.9-fold (OR = 6.93, *P* = 0.037) and, for each 1-mm decrease in the RMS, the risk of re-contact increased by 33% (0.67, 0.035).

Healing processes among DB, DM and DL sites might have been different because of three different causes of bony defect. First, the periodontal condition at the DL site was much better than that at DM and DB sites. Periodontal healing at the DL site showed much less relevance than that at DM and DB sites (Fig. 2). DBD depth at the DL site (1.91 ± 1.19 mm at Po.6 m) was in accordance with the periodontal-healing standard that alveolar-bone height must be restored to within 2 mm of the CEJ [35]. Periodontal healing here could be characterized as normal healing of alveolar sockets. Second, defects at DM and DB sites were much deeper than those at the DL site (Table 1), and might have been caused by M3M growth. Chen studied 421 CBCT images of M3Ms and found that 96.2% of M3Ms were at median or buccal sides distal to M2Ms [36]. Conversely, we found that PD reduction could be due more to soft-tissue variation than hard-tissue variation (Table 4). Most participants in the present study were asymptomatic, and a thickness of soft tissue > 5 mm at DM and DB sites (difference in reduction of DBD depth and PD reduction is shown in Table 1) would be considered to be the long junctional epithelium [16]. Also, the lack of keratinized gingival coverage might account for the vulnerability of the M3M region to periodontal diseases [37]. It would be better to prevent reduced growth of the epithelium and improve periodontal healing of the M2M with guided tissue regeneration [35]. Third, a defect at the DB site is also created (at least in part) by iatrogenic removal of buccal bone during M3M surgery [12]. A significant correlation between reduction in DBD depth and PD reduction was found between DM and DB sites (Fig. 2). However, the percentage of severe bone loss (DBD depth ≥ 4 mm) was greater at the DB site (72.8% at baseline, 45.1% at Po.6 m) than that at the DM site (63.4% at baseline, 27.7% at Po.6 m) (Table 1).

Healing of a periodontal defect is based on recovery of surrounding alveolar bone [35]. Previous periodontal probing and 2D radiographs are not sufficient to evaluate morphology of bone pockets, which is critical for prediction of periodontal healing [16]. Furthermore, bone height does not to indicate the stability of periodontal healing. A more accurate and predictable index in three dimensions is necessary.

We proposed, for the first time, the volume of bone regenerated in the distal 2 mm of the M2M to be such an index. This parameter reflects the minimum thickness of bone required to prevent resorption of surrounding bone, and denotes the stability of bone distal to the M2M. Our postulation is based on two main observations. First, Porto reported, in an anatomic study, that the minimum thickness of stable alveolar bone in posterior teeth is 1.98 ± 1.33 mm [38]. Second, it is widely accepted that 2 mm of alveolar width around the implant is the minimum volume to prevent further bone resorption [39]. When using digital software to measure bone volume, the error between the measured value and real value is relatively small. According to a study by Liu and colleagues [18], the deviation was < 7%. Therefore, the accuracy of our new 3D index (the volume of bone regenerated in the distal 2 mm of the M2M) was sufficient for clinical application. The morphology of bone pockets is presented in Fig. 1g. A significantly high correlation was found between the volume of bone regenerated in the distal 2 mm and reduction in the DBD depth (*r* = 0.74, *P*<0.001). In addition, all statistical analyses involving these two factors revealed similar results, which provides further evidence of the effectiveness of this new index. Conversely, the volume of bone regenerated in the distal 2 mm showed a weak correlation with the total volume of bone regenerated in the alveolar socket (*r* = 0.20, *P* = 0.004) whereas reduction in DBD depth did not (*r* = − 0.01, *P* = 0.871). Our new index might bridge the healing of the alveolar socket of the M3M and repair of distal periodontal tissue of the M2M.

Our study had two main limitations. First, a control group (total extraction of the M3M) was not included. The reason was because coronectomy is widely accepted as an effective alternative to prevent IAN injury [2]. Also, our ethics committee suggested that random grouping might not suitable in our study. Second, age is a critical factor for DBD healing [29], but the patients recruited for our study were relatively young.

## Conclusions

The DBD depth at baseline was the most influential factor for healing of a DBD of the M2M after M3M coronectomy. The remaining enamel and a small RMS could contribute to re-contact of the root complex and the M2M. The volume of bone regenerated in the distal 2 mm of the M2M was an accurate 3D indicator reflecting the stability of distal periodontal healing of the M2M.

## Data Availability

The datasets used and analyzed during the current study are available from the corresponding authors upon reasonable request.

## Abbreviations

3D: Three-dimensional
2D: Two-dimensional
BL: Baseline, the day after surgery
CBCT: Cone-beam computed tomography
CEJ: Cementoenamel junction
DB: Disto-buccal
DM: Disto-middle
DL: Disto-lingual
DBD: Distal intra-bony defect
DMR: Distal marginal ridge
IAN: The inferior alveolar nerve
M2M: Mandibular second molar
M3M: Mandibular third molar
PD: Probing depth
Po. 6 m: 6 months postoperatively
RMS: Retromolar space
